# Seasons in the sun: the impact on IVF results one month later

**Published:** 2016-06-27

**Authors:** F Vandekerckhove, H Van der Veken, K Tilleman, I De Croo, E Van den Abbeel, J Gerris, P De Sutter

**Affiliations:** Fertility Center, University Hospital, Gent, Belgium.

**Keywords:** Weather conditions, live birth rate, ovarian stimulation, oocyte maturation, vitamin D, melatonin

## Abstract

**Background:**

Several retrospective studies have evaluated seasonal variations in the outcome of IVF treatment. Some also included weather conditions, mostly temperature and hours of daylight. The results were conflicting.

**Methods:**

In a retrospective study we analysed all fresh cycles (N = 9865) that were started between January 1, 2007 and December 31, 2012. Because some patients were included more than once, correction for duplicate patients was performed. We focused on individual variables provided as monthly results by our national meteorological institute. We evaluated if weather conditions determined by temperature, rain and sunshine at the start of ovarian stimulation had an effect on the outcome of IVF in terms of number of mature and fertilized oocytes, pregnancy and live birth rates. We shifted the results in IVF outcome to the weather results of one month earlier, as we supposed that the selection of good quality oocytes might start in the weeks before ovarian stimulation is initiated.

**Results:**

There was a clear trend towards better results when the “early” weather conditions (one month before the treatment cycle) were good. There was a statistically significant correlation between the number of rainy days (Pearson Correlation -0.326; p < 0.01) and the rain flow (Pearson Correlation -0.262; p < 0.05) on the one hand and the live birth rate per cycle on the other.

The live birth rate per cycle was statistically different between cohorts of patients that were stratified into quartiles of sunshine hours (p < 0.01) and of number of rainy days (p < 0.05) during the month before the start of ovarian stimulation.

**Conclusions:**

Weather conditions during the month before IVF treatment have an impact on live birth rate.

## Background

It is well known that seasons have an influence on spontaneous fecundity in animals. The effect of seasonality on mammalian reproduction has been clearly demonstrated already (Wood et al., 2006). Sexual activity in humans is not bound to seasons (Levine et al., 1990; Udry and Morris, 1967) although variations in natural conception and birth rates exist (Becker, 1981; Boklage, Zincone and Kirby, 1992; Ehrenkranz 1983; Lam and Miron, 1991; Odegard, 1977; Roenneberg and Aschoff, 1990; Rojansky, Brzezinski and Schenker, 1992; Rosenberg, 1966; Mathers and Harris, 1983).

Conflicting reports on the impact of seasons on human in vitro fertilization (IVF) have been published. Some studies grouped their patients into seasons as defined by four three-month periods (Braga et al., 2012; Revelli et al., 2005; Rojansky et al., 2000; Stolwijk et al., 1994; Wunder et al., 2005) or by two six-month periods (Wood et al., 2006). The date to include patients into different seasonal groups also differed among protocols: some used the date of triggering oocyte maturation (Revelli et al., 2005) while others referred to the start of the stimulation (Rojansky et al., 2000; Wunder et al., 2005). The outcome variables were defined as rates for fertilization, pregnancy and implantation but no study reported on live birth rates per cycle. All outcome variables were plotted against the time period in which the actual IVF procedure was carried out. Only one study also included individual weather conditions such as temperature, humidity, light hours and sun hours in its analysis (Rojansky et al., 2000).

We chose a different approach for our analysis. Morphological and endocrine studies have shown that antral follicles are present throughout the cycle and are continuously available for stimulation by FSH. It is well known that folliculogenesis takes some 13 months for the primordial follicles to become antral follicles. This means that the influence of temporary external factors such as weather conditions during this period on oocyte development is difficult to evaluate. At the end of the luteal phase, the largest visible follicles are 2-5 mm in size (Chikazawa, Araki and Tamada, 1986; Gougeon, 1986; Mcnatty et al., 1983). These follicles will grow under the influence of FSH during the next cycle (Baird, 1990; Hodgen, 1982). The number of follicles that is available in this “luteo-follicular transition” is 5-10 per ovary (Gougeon, 1996; Hodgen, 1982; Pache et al., 1990). These are the follicles that will start to grow under the influence of exogenous FSH in a treatment cycle. Epigenetic and other environmental factors are becoming more and more important in the reproductive pathway (Crujeiras and Casanueva, 2015). It is plausible that external factors during the month before may have an impact on the quality of the oocytes that are harvested for IVF one month later. Various weather conditions may be the factors involved. For this reason we studied the impact of sunshine, rain and temperature on IVF outcome the same month and one month later.

To evaluate the impact of the same weather conditions on spermatogenesis, we also repeated our analysis with the earlier weather results of 2 months beforehand.

## Methods

In this retrospective study we analysed all fresh IVF/ICSI cycles that were started between January 1, 2007 and December 31, 2012 in our university centre. The inclusion date was the start of the gonadotropin stimulation. Because we wanted to study the impact of weather conditions on IVF outcome, only Belgian patients were included. From 2013 onwards we gradually introduced blastocyst transfer in our routine practice, which had a profound positive effect on our results. We omitted data later than 31/12/2012 to avoid bias from this change. The study was approved by the ethics committee of our university (ref. EC/2015/1332).

IVF and ICSI cycles were both included and various protocols for controlled ovarian hyperstimulation were applied. Either recombinant FSH (Gonal F®, Merck Serono, Darmstadt, Germany) or urinary FSH (Menopur®, Ferring Pharmaceuticals, Saint-Prex, Switzerland) was used with daily doses between 150 and 300 U, dependent on age, anti-Mullerian hormone (AMH) levels and previous response if applicable. In the agonist group triptorelin (Decapeptyl®, Ipsen, Paris, France) 0.1 mg was administered subcutaneously for 7 days from cycle day 1 on and gonadotropins were started on cycle day 3. In the antagonist group a fixed protocol was used: gonadotropins were started on cycle day 3 and cetrorelix (Cetrotide®, Merck Serono, Darmstadt, Germany) 0.25 mg was injected subcutaneously as a daily dose from the 6th day of stimulation until the day of oocyte maturation triggering. The monitoring procedure was performed as described elsewhere (Vandekerckhove et al., 2014) and 5000 U of human chorionic gonadotropin (hCG) (Pregnyl®, Merck Sharp & Dohme, New Jersey, United States) was used as a maturation trigger.

Embryo transfer was performed on day 3 and according to the Belgian legislation on the maximum number of embryos to be transferred (De Neubourg et al., 2013).

Monthly weather conditions were extracted from the website of our national meteorological institute (Koninklijk Meteorologisch Instituut van België). For all months between December 1, 2006 and January 31, 2013 reliable data could be obtained on temperature (°C), rain (L/m2), number of rainy days (days) and sunshine hours (Hrs) (Table I). We calculated the number of mature oocytes, the number of normally fertilized oocytes, the pregnancy rate and the live birth rate per started cycle, according to international standards (Zegers-Hochschild et al., 2009). Results were expressed as monthly means, which allowed us to compare them with the monthly weather results.

**Table I:** — Meteorological data.

Month	Temperature (°C)	Rain (L/m2)	Rainy days (days)	Sunshine (hours/month)
Nov/06	9.1	71.6	20	79
Dec/06	5.9	93.0	18	40
Jan/07	7.2	82.3	26	35
Feb/07	6.8	95.4	18	49
Mar/07	8.0	61.9	20	151
Apr/07	14.3	0.0*	0*	301
May/07	14.6	103.4	21	167
Jun/07	17.5	99.2	19	134
Jul/07	17.2	96.7	18	173
Aug/07	17.1	56.9	13	150
Sep/07	14.1	57.6	16	110
Oct/07	10.4	65.2	11	115
Nov/07	6.8	71.7	22	56
Dec/07	4.1	89.2	19	77
Jan/08	6.5	70.7	23	48
Feb/08	6.1	35.4	11	131
Mar/08	6.3	140.5	24	73
Apr/08	9.3	45.8	14	150
May/08	16.4	53.9	11	237
Jun/08	16.1	69.9	17	189
Jul/08	18.0	101.9	20	183
Aug/08	17.6	89.3	19	136
Sep/08	14.0	70.8	12	157
Oct/08	10.5	72.4	19	122
Nov/08	6.9	67.6	26	39
Dec/08	2.8	43.3	13	66
Jan/09	0.7	62.9	12	89
Feb/09	3.6	57.1	20	33
Mar/09	6.7	68.2	15	150
Apr/09	12.5	47.1	15	196
May/09	14.4	43.1	17	196
Jun/09	16.5	64.5	11	225
Jul/09	18.7	73.1	20	213
Aug/09	19.4	34.7	9	258
Sep/09	15.8	29.1	10	156
Oct/09	11.3	105.0	17	90
Nov/09	9.7	98.0	25	48
Dec/09	2.9	80.8	19	46
Jan/10	0.1	43.8	18	49
Feb/10	2.5	76.1	24	29
Mar/10	6.7	50.2	13	118
Apr/10	10.3	15.7	6	223
May/10	11.2	66.6	14	172
Jun/10	17.4	30.0	9	259
Jul/10	20.5	62.8	13	252
Aug/10	17.0	187.4	23	136
Sep/10	14.2	109.8	18	143
Oct/10	10.6	70.8	16	119
Nov/10	6.1	124.7	21	24
Dec/10	-0.7	76.2	26	33
Jan/11	4.0	90.5	21	52
Feb/11	5.4	44.0	15	55
Mar/11	7.7	22.4	7	204
Apr/11	14.1	25.8	11	239
May/11	14.8	22.5	9	264
Jun/11	16.8	72.3	19	181
Jul/11	16.0	55.6	20	140
Aug/11	17.3	189.3	22	145
Sep/11	16.5	83.1	10	173
Oct/11	12.1	48.8	16	162
Nov/11	8.6	8.5	11	115
Dec/11	6.1	152.1	26	52
Jan/12	5.1	86.4	23	49
Feb/12	0.7	30.0	16	95
Mar/12	8.9	32.9	8	166
Apr/12	8.4	104.1	21	113
May/12	14.3	63.4	14	190
Jun/12	15.4	133.1	21	147
Jul/12	17.3	115.7	18	173
Aug/12	19.2	22.5	12	219
Sep/12	14.5	51.6	12	175
Oct/12	11.1	119.4	21	120
Nov/12	7.1	44.7	18	50
Dec/12	5.1	172.7	28	31
Jan/13	2.1	53.6	19	36

* Data checked by phone call to the meteorological institute.

Firstly bivariate correlation was performed by linear modelling between monthly weather conditions and IVF results. Secondly the same IVF outcome variables were plotted against the quarter of the year and against the weather results stratified per quartile for each individual meteorological variable (Kruskal-Wallis Test).

Afterwards we shifted the results in IVF outcome to the weather results of one month earlier and one month later. Pregnancy and live birth rates in IVF treatment are dependent on several factors. Because of the retrospective design of our study and the long time period of inclusion, a more detailed multivariate analysis would have been desirable. Our data were insufficient for this purpose.

All statistical tests were performed at the 5% significance level with SPSS V22.

## Results

We included 9865 cycles from 4259 individual patients in our study.

The mean age of the patients was 32.4 years (Table II). Female subfertility was diagnosed in 26.6% of the cases, a male factor was found in 30.7% and a mixed female/male reason in 10.5%. 25.4% of the cases were included as unexplained infertility.

**Table II:** — Descriptive statistics.

Variable	
Number of cycles	9865
Number of patients	4259
Female age (years)	32.4 (4.9)
Diagnosis Female Male Female and male Unexplained infertility	26.6% 30.7% 10.5% 25.4%
ICSI or IVF ICSI IVF	74.0% 26.0%
Stimulation protocol Agonist Antagonist	90.6% 9.4%
Number of mature oocytes	9.1 (7.1)
Number of normally fertilized oocytes	6.3 (5.4)
Pregnancy rate/cycle	26.7%
Live birth rate/cycle	16.2%

Numbers are expressed as means (SD) unless explained differently.

An agonist protocol for ovarian stimulation was used in 90.6% of the cycles. In the more recent years, the use of an agonist was getting more popular, especially in patients with a good ovarian reserve.

We harvested a mean of 9.1 mature oocytes and obtained 6.3 normally fertilized oocytes per cycle.

The pregnancy rate was 26.7% per cycle and the live birth rate for the whole series of data was 16.2%. These outcome data were from the period before 2013. We then gradually changed from day 3 to day 5 transfers and all outcome variables have much improved since that time.

The monthly results were plotted on a time axis. The number of mature oocytes, the number of fertilized oocytes, the pregnancy rate and live birth rate showed no differences over time as evaluated per month.

We then stratified the outcome variables per quarter of the year (January-March, April-June, July-September and October-December). A Kruskal-Wallis Test revealed no differences again (p > 0.05) for each individual variable.

Afterwards we focused on the impact of monthly weather conditions on IVF outcome. By linear modelling we tested for correlation between IVF outcome and the various weather conditions. If we compared the weather results to the IVF outcome of a cycle where the gonadotropin stimulation was started the same month, all results were inconsistent. There was no link whatsoever. If we used the weather data of one month earlier however, a trend towards better IVF results with good weather conditions appeared (Table III). There was a significant association between rain flow and live birth rate (p 0.026) and between the number of rainy days and live birth rate (p 0.005) as well.

**Table III:** — Correlation between early weather conditions and IVF results.

		Mature oocytes	Fertilized oocytes	Pregnancy rate/cycle	Live birth rate/cycle
E-Temperature (°C)	Pearson Correlation	0.057	-0.041	0.056	0.126
	Sig. (2-tailed)	0.634	0.735	0.639	0.29
E-Rain (L/m^2^)	Pearson Correlation	-0.118	-0.139	-0.120	-0.262*
	Sig. (2-tailed)	0.324	0.245	0.317	0.026
E-Rainy days	Pearson Correlation	-0.072	-0.078	-0.182	-0.326**
	Sig. (2-tailed)	0.548	0.517	0.126	0.005
E-Sunshine (Hrs)	Pearson Correlation	0.014	-0.014	0.150	0.228
	Sig. (2-tailed)	0.905	0.906	0.207	0.054

* Correlation is significant at the 0.05 level (2-tailed).

** Correlation is significant at the 0.01 level (2-tailed).

**Fig. 1:**
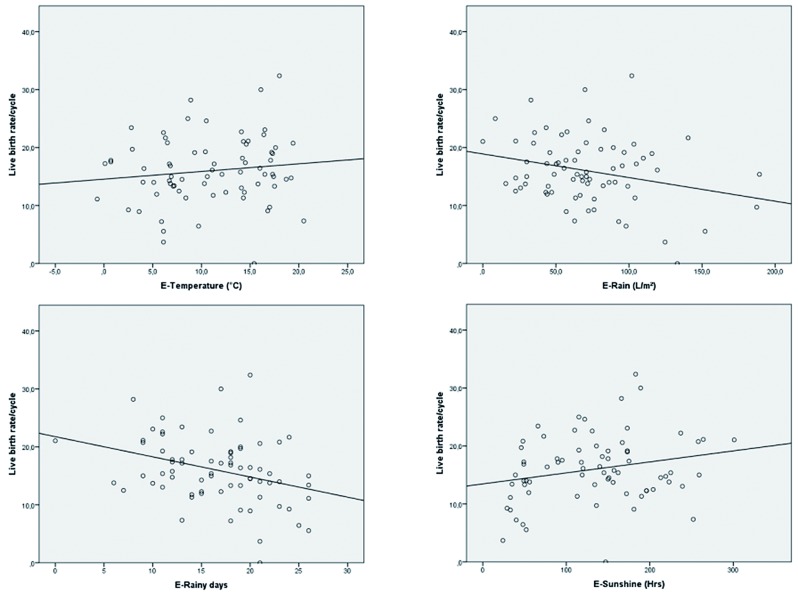
— Association between early weather conditions and live birth rate per started cycle

Results for live birth rate are presented in detail in Figure 1.

If the same procedure was performed again with the weather conditions of one month after or two months before the start of the stimulation procedure, all consistency disappeared again.

**Fig. 2:**
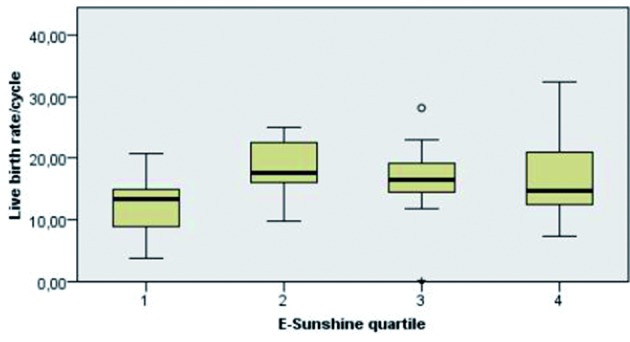
— Differences in live birth rate per quartile of sun exposure one rnonth before treatment.

**Fig. 3:**
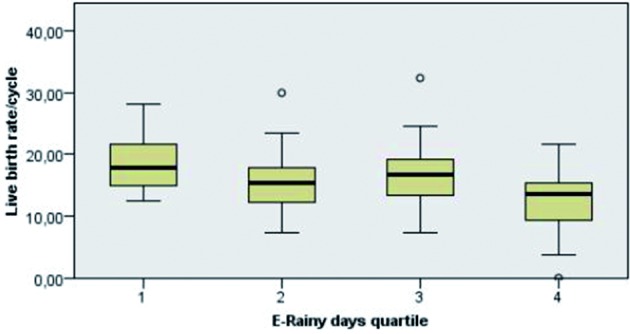
— Differences in live birth rate per quartile of rainy days one month before treatment.

Results for temperature, rain, number of rainy days and sunshine hours were grouped together into quartiles (Table IV). A Kruskal-Wallis Test was performed to evaluate if any differences were noted for the following outcome variables: number of mature oocytes, number of normally fertilized oocytes, pregnancy rate and live birth rate. The procedure was done in “real time” and repeated for “early weather conditions” (one month before) and “late weather conditions” (one month later). A statistically significant difference in live birth rate (p 0.005) was noted between quartiles of “early” sunshine hours (Fig. 2), especially between quartile 1 and 2 (p 0.003). Numbers (mean +/- SD) for live birth rate per cycle were 12.4 +/-4.8, 18.4 +/- 4.4, 16.6 +/- 5.8 and 16.6 +/- 6.6 in quartiles 1 to 4 respectively. The live birth rate also varied significantly (p 0.015) between quartiles of the number of “early” rainy days, most notable between quartile 1 and 4 (p 0.008). The live birth rates per ascending quartile (mean +/- SD) are illustrated in Figure 3. They were 18.6 +/- 4.5, 16.3 +/- 5.5, 16.7 +/- 5.9 and 12.3 +/- 5.9 respectively.

**Table IV:** — Quartiles of montlhly weather conditions.

		Temperature (°C)	Rain (L/m’)	Rainy days	Sunshine (Hrs)
**Mean**		10.9	72.4	16.7	133.6
Median		10.9	67.9	17.5	138.0
**Percentiles**	25	6.6	45.0	12.0	58.5
	50	10.9	67.9	17.5	138.0
	75	16.1	94.2	21.0	179.5

Finally the effect of possible confounders such as female age, male factor infertility and the percentage of embryo transfers at the blastocyst stage was checked: they were all equally distributed (p > 0.05) among the quartiles of the 4 weather variables (Kruskal-Wallis Test).

## Discussion

The fertilization rate was constant over the years and also not influenced by seasons or weather conditions. This was not confirmed by Rojansky et al. (Rojansky et al., 2000), who described higher fertilization rates and good quality embryo rates with extended light hours. Pregnancy rates were not influenced by light in his study.

Up to our knowledge, our study was the first to include live birth rates per cycle as an outcome variable. We were also the first to shift the outcome variables to the weather conditions of one month before. We suppose that the higher live birth rate with more sun exposure and lower rain flow and number of rainy days had to do with the quality of the oocytes that were selected for maturation through the stimulation procedure.

### Melatonin

Melatonin is secreted by the pineal gland, which is located at the backside of the third ventricle. In animals it suppresses the functionality of the hypothalamo-pituitary-gonadal axis. Its secretion is stimulated by darkness and inhibited by light, which explains seasonal variations in gonadal activity. A diurnal rhythm has been found in men as well. But how does it work in IVF? Melatonin exhibits antioxidant activity (Galano, Tan and Reiter 2011). In IVF high free radical levels surround growing zygotes. If melatonin was added in bovine IVF culture medium, it had no effect on cleavage and blastocyst percentage (Cheuqueman et al., 2015). In the mouse model however, it seemed to have a protective effect (Dehghani-Mohammadabadi et al., 2014). In humans it is known that pre-ovulatory follicular fluid contains higher concentrations of melatonin than serum. Granulosa cells in mature follicles secrete it as well. In patients with polycystic ovarian syndrome (PCOS), in vitro maturation of oocytes (IVM) is sometimes performed before IVF. If melatonin was added to the IVM culture medium, it improved the IVM procedure (cytoplasmic maturation) and clinical outcomes (Kim et al., 2013). IVF outcome was also compared in patients with sleeping disturbances who were administered melatonin or not. The oocyte and embryo quality improved in the treatment group (Eryilmaz et al., 2011). In a randomized controlled trial of patients with known low oocyte quality, co-administration of melatonin with myo-inositol and folic acid resulted in better oocyte quality and pregnancy outcome (Rizzo, Raffone and Benedetto, 2010).

These findings do not really offer an explanation for our results: we found that live birth rates were higher if the patients were exposed to more sunshine hours the month before treatment. They probably had lower levels of melatonin at that time.

### Vitamin D

The association between sun exposure and Vitamin D status is well known, as confirmed again recently in Australia (Pittaway et al., 2013). If we are looking for an explanation of how sun exposure can influence IVF outcome, Vitamin D must be evaluated as an intermediate factor. A large prospective trial to evaluate the impact of diet, including Vitamin D supplements, on IVF outcome was recently started (Kermack et al., 2014). The Vitamin D status was unrelated to IVF outcome in a retrospective study, which included only euploid embryos at blastocyst stage (Franasiak et al., 2015). This was not confirmed by Polyzos et al. (Polyzos et al., 2014) who concluded that Vitamin D deficiency, defined as a serum level of 25-OH vitamin D < 20 ng/mL, was independently associated with lower clinical pregnancy rates. Both studies were about blastocyst transfers, which was not the case in our series. Firouzabadi et al. (Firouzabadi et al., 2014) found no correlation between Vitamin D levels and pregnancy rates and only included embryo transfers at multicellular stage. There also seem to exist differences between races: especially in non- Hispanic whites, pregnancy rates were lower with lower levels of Vitamin D (Rudick et al., 2012).

Vitamin D and sun exposure are related, but the link between sunshine hours and live birth rates as we found it in our study is not completely explained in this way.

Although melatonin and vitamin D may have a special role in folliculogenesis , the results of current studies are contradictory and thus insufficient to provide a guidance regarding a potential relationship between vitamin D or melatonin on the one hand and the quality of oocytes in response to ovarian stimulation on the other hand. The link between weather conditions the month before treatment and the outcome of IVF treatment remains a mystery for the moment.

### No effect on spermatogenesis

Knowing that sperm quality may be influenced by external factors during spermatogenesis, that takes at least 2 months, we also checked if our primary outcome variables were influenced by weather conditions more than 1 month before treatment. At that time we found no correlation between outcome variables and weather conditions 2 months beforehand. Because no correlation was found between IVF outcome and the weather results of 2 months before treatment, we concluded that spermatogenesis was not influenced at all.

## Conclusions

We found a link between weather conditions during the month before IVF treatment and the live birth rate per cycle. We solely focused on weather conditions. As we supposed that sunshine exposure was the external factor that might have improved the quality of the eggs selected to grow, we might as well suppose that a lot of other external factors “the month before” can be important for success. So further studies on the impact of other external or environmental factors are mandatory.

## Abbreviations

AHM: anti-Mullerian hormone
FSH: follicle-stimulating hormone
hCG: human chrorionic gonadotropin
ICSI: intracytoplasmatic sperm injection
IVM: in vitro maturation
IVF: in vitro fertilization
PCOS: polycystic ovary syndrome
